# Whole exome sequencing of a consanguineous family identifies the possible modifying effect of a globally rare *AK5* allelic variant in celiac disease development among Saudi patients

**DOI:** 10.1371/journal.pone.0176664

**Published:** 2017-05-15

**Authors:** Jumana Yousuf Al-Aama, Noor Ahmad Shaik, Babajan Banaganapalli, Mohammed A. Salama, Omran Rashidi, Ahmed N. Sahly, Mohammed O. Mohsen, Harbi A. Shawoosh, Hebah Ahmad Shalabi, Mohammad Al Edreesi, Sameer E. Alharthi, Jun Wang, Ramu Elango, Omar I. Saadah

**Affiliations:** 1 Department of Genetic Medicine, Faculty of Medicine, King Abdulaziz University, Jeddah, Saudi Arabia; 2 Princess Al-Jawhara Al-Brahim Center of Excellence in Research of Hereditary Disorders, King Abdulaziz University, Jeddah, Saudi Arabia; 3 Department of Biological Sciences, Faculty of Science, King Abdulaziz University, Jeddah, Saudi Arabia; 4 Department of Pediatrics, King Fahd Armed Forces Hospital, Jeddah, Saudi Arabia; 5 Primary Healthcare Center, Ministry of Health, Jeddah, Saudi Arabia; 6 Division of Gastroenterology, Department of Pediatrics, Dhahran Health Center, Saudi Aramco Medical Services Organization, Dhahran, Saudi Arabia; 7 Division of Pediatric Gastroenterology, Department of Pediatrics, Faculty of Medicine, King Abdulaziz University, Jeddah, Saudi Arabia; Huashan Hospital Fudan University, CHINA

## Abstract

Celiac disease (CD), a multi-factorial auto-inflammatory disease of the small intestine, is known to occur in both sporadic and familial forms. Together HLA and Non-HLA genes can explain up to 50% of CD’s heritability. In order to discover the missing heritability due to rare variants, we have exome sequenced a consanguineous Saudi family presenting CD in an autosomal recessive (AR) pattern. We have identified a rare homozygous insertion c.1683_1684insATT, in the conserved coding region of *AK5* gene that showed classical AR model segregation in this family. Sequence validation of 200 chromosomes each of sporadic CD cases and controls, revealed that this extremely rare (EXac MAF 0.000008) mutation is highly penetrant among general Saudi populations (MAF is 0.62). Genotype and allelic distribution analysis have indicated that this *AK5* (c.1683_1684insATT) mutation is negatively selected among patient groups and positively selected in the control group, in whom it may modify the risk against CD development [p<0.002]. Our observation gains additional support from computational analysis which predicted that Iso561 insertion shifts the existing H-bonds between 400^th^ and 556^th^ amino acid residues lying near the functional domain of adenylate kinase. This shuffling of amino acids and their H-bond interactions is likely to disturb the secondary structure orientation of the polypeptide and induces the gain-of-function in nucleoside phosphate kinase activity of *AK5*, which may eventually down-regulates the reactivity potential of CD4^+^ T-cells against gluten antigens. Our study underlines the need to have population-specific genome databases to avoid false leads and to identify true candidate causal genes for the familial form of celiac disease.

## Introduction

Celiac disease (CD) [MIM 212750] is an intestinal autoimmune disorder caused by permanent intolerance to gluten fraction of wheat, barley, and rye [1]. CD may occur in any age group, either asymptomatically (in children and adults), minimally symptomatic or in severe form (mostly seen in children) [2]. The overall prevalence of CD in people of European ancestry is between 0.5% to 1% [3]. The diagnosis of CD is established by a combination of clinical examinations, serological tests (anti-tissue transglutaminase and anti-endomysium (EMA) auto-antibodies and histological analysis of small bowel biopsies. Until last decade CD is widely believed to be non-existing in the Middle East, however, due to advances in diagnostic methods, it is starting to be increasingly seen in Saudi population, who consume wheat as the major dietary staple food [4].

Celiac disease has a complex etiology in which both genetic and environmental factors play a determinant role in the disease onset. The genetic liability of CD is evidenced through its high prevalence rate in first-degree relatives of patients, as well as because of its strong association with class II alleles (DQ2 or DQ8 haplotypes) of human leukocyte antigens (HLA). It is noteworthy, that up to 95% of CD patients carry HLA- DQ2.5 heterodimer (encoded by DQA1*05 and DQB1*02) alleles, while the remaining 5% have HLA-DQ8 (HLA-DQA1*03 and HLA-DQB1*03:02) alleles [5]. Almost 100% individuals with CD are positive to either HLA DQ2 or DQ8 alleles [6]. Although around 10–30% general population is positive to HLA-DQ 2.5 (20–30%) and HLA-DQ8 (10%) heterodimers, only 3% of them are known to develop gluten intolerance [7]. This implies that the inheritance of at least one of HLA-DQ2 or HLA-DQ8 alleles can be regarded as an almost necessary factor but alone do not determine the development of CD. This suggests that besides HLA class II genes, additional non-HLA genes/loci are also having a major contribution in developing celiac disease.

The introduction of genome wide association studies (GWAS) has been a significant breakthrough in discovering the definite associations of 39 non-HLA loci for celiac disease [8] [9] [10]. However, some of these susceptibility loci are shared between different auto-immune disorders like celiac disease, Rheumatoid Arthritis (RA) [11] and Crohn’s [12]. Together these 42 non-HLA loci may only explain 10% of the actual celiac disease risk [13]. Recent reanalysis of immunochip data of large number of CD cases revealed 5 more new immunity function related loci [14]. The combinational effect of HLA and non-HLA genetic loci is estimated to account for only 50% of the CD heritability, leaving the remaining 50% heritable factors unknown [15]. Although, GWAS studies have successfully identified the key genetic pathways (based on the frequency of common genetic variants) contributing to celiac disease phenotypes, so far they failed in identifying causal genes [16]. Moreover, majority of the CD-GWAS were conducted in European patients, missing population-specific genetic effects in other ethnic groups such as Arabs. Additionally, genetic linkage studies are also not successful in identifying the disease causal genes for CD [17].

Although most of the molecular information of CD genetics has come from sporadic cases, it did not lead to the identification of the causal genes [18]. Given the recent indications that celiac disease is often inherited in some families [from Europe and the Middle East], co-segregation of CD specific multiple genetic loci in celiac family members is expected [19]. In this context, whole exome sequencing presented us with an opportunity to examine a consanguineous family to reveal the causal mutation for Celiac disease. Saudi Arabia, being the global epicenter of the consanguinity (>56% of all marriages are between 1^st^ cousins), has a higher incidence of newborns with autosomal recessive disorders [20]. Despite the increasing prevalence, genetic investigations carried out on Saudi CD patients are few [21]. Therefore, present study has aimed to discover the causal gene for CD by whole exome sequencing of a consanguineous family from Saudi Arabia.

## Material and methods

### Recruitment of celiac family, sporadic cases, and controls

Ethics approval for the present study was granted by the Institutional Ethics Committee for Human Research, King Abdulaziz University Hospital (KAUH), Jeddah. From our pediatric gastroenterology clinic, we have recruited one consanguineous family presenting an autosomal recessive form of CD with two affected sibs. All members of this family were thoroughly screened by consultant gastroenterologists for the clinical evidence of CD. The initial celiac disease diagnosis was made by gliadin and endomysium (EMA) or tissue transglutaminase (TGA) antibody detection, followed by confirmatory small bowel biopsy as per the criteria set out by European Society for Pediatric Gastroenterology, Hepatology and Nutrition (ESPHGAN) [22]. A total of 100 sporadic CD cases in addition to 100 healthy control volunteers were also recruited. The controls recruitment was based on their self-health reports and later by clinical and serological confirmations.

### Clinical sampling

Five mL of peripheral blood sample was collected for genetic analysis from each participant, after obtaining their signed informed consent from patients or their parents (for minors).

### DNA isolation

The genomic DNA isolation from peripheral blood was carried out with QIAamp DNA blood Kit (C# 51104) as per the manufacturer’s instructions. NanoDrop ND-1000 UV- VIS Spectrophotometer was used to assess DNA concentration and purity. Agarose gel electrophoresis was done to assess both DNA intactness and the average molecular weight of each sample.

### Molecular genetic analysis

We have used the CD affected autosomal recessive family as our discovery cohort, whereas sporadic CD cases and healthy controls as the validation cohort. Our experimental strategy is as follows. At first, we exome sequenced selected members in a CD family (2 probands + 1 healthy sibling + parents) and then validated (by dideoxy sequencing) our findings in the other family members, sporadic cases, and controls.

### Exome sequencing

The blood genomic DNA (100 ng/μl) was utilized to prepare whole exome-enriched library using a SureSelect solution-based capture reagent (Agilent Technologies, USA), which traps exonic regions of protein-coding genes listed in CCDS and RefSeq databases [23] [24] [25] [26]. The 100-bp library was sequenced on the HiSeq2000 platform (Illumina), and read alignment was done against the human chromosome reference assembly build 38 (GRC38, NCBI) using BLAST (version 0.6.4d) [27], followed by base quality recalibration with the GATK tool [28]. The alignment of 15 GB of sequencing data (from each sample) represented that up to 87% of bases were covered by 100X. SAM tools were used for calling SNPs and Indels [29]. Variants which showed high-quality Phred scoring of at least 40 (or 30 if observed on both strands) were included for further analysis. Common mutations (>1% frequency) were excluded based on the information available in dbSNP130 [30], The Exome Aggregation Consortium (ExAC) databases and 1000 Genomes Pilot 1 datasets [31]. The annotation of novel mutations (like splice site, indel, nonsense, and missense) was done with help of ANNOVAR software [27]. Functional consequences of non-synonymous mutations were predicted using the SIFT [32] and PolyPhen-2 [33] and Mutation Taster algorithms [34].

### Targeted sequencing, sequence alignment, and mutation identification

The first set of exome sequence derived CD- causal (suspected) mutations were further screened in our study participants by the dideoxynucleotide sequencing method. In brief, we initially designed oligonucleotide primers (using an open source software PRIMER3; http://frodo.wi.mit.edu/) flanking rare alleles (minor allele frequency of 0.01) before carrying out standard 35 cycle PCR amplifications. The success of all PCR reactions was confirmed on an agarose gel, before purifying and cycle sequencing them in ABI-Prism 3700 Genetic Analyzer. The list of all analyzed loci and primers can be found as S1 Table. The BioEdit (version 6.0.7) program was used to align and annotate nucleotide sequence mismatches.

### DNA sequence conservation and protein structure analysis

To examine the nucleotide sequence conservation pattern of *AK5* gene across related species (8 primates), we have performed multiple sequence alignment using Ensembl browser (www.esembl.org). The correlation between *AK5* c.1683_1684insATT genotype versus its protein phenotype was explored utilizing multidimensional computational approaches. Initially, PDB sourced 3-dimensional structure of *AK5* protein was used as template (PDBID: 2BWJ) for creating mutant protein versions through homology modeling. Then, the energy minimization step for full-length wild-type and mutant protein structures was done using NOMAD-Ref Server [35]. In next stages, the amino acid substitution induced structural disturbances in *AK5* protein molecule was measured through a computational (Cα traces and backbone atoms) superimposition of wild-type and mutant protein structures and by Pymol based root mean square value calculation (RMSD) among equivalent atoms. The whole structure level divergence of mutant proteins was considered to be positive when its RMSD score fall <2.0 Å, whereas, for amino acid structure level divergence minimum RMSD score considered was < 0.2 Å.

### Statistical analysis

Statistical analyses were performed with GraphPad QuickCalcs, version 6.0 (GraphPad Software Inc., USA), online software. Genotype and allele frequencies were presented in the form of percentages (%). Statistically significant difference in allele and genotype frequencies was determined by Pearson’s standard chi-squared test, odds ratio (OR), and 95% confidence interval (CI). Hardy-Weinberg equilibrium (HWE) was tested using the χ2 test for goodness of fit, and a p value < 0.05 was considered a statistically significant disequilibrium.

## Results

### Clinical presentation of CD patients in the family

**Fig 1:**
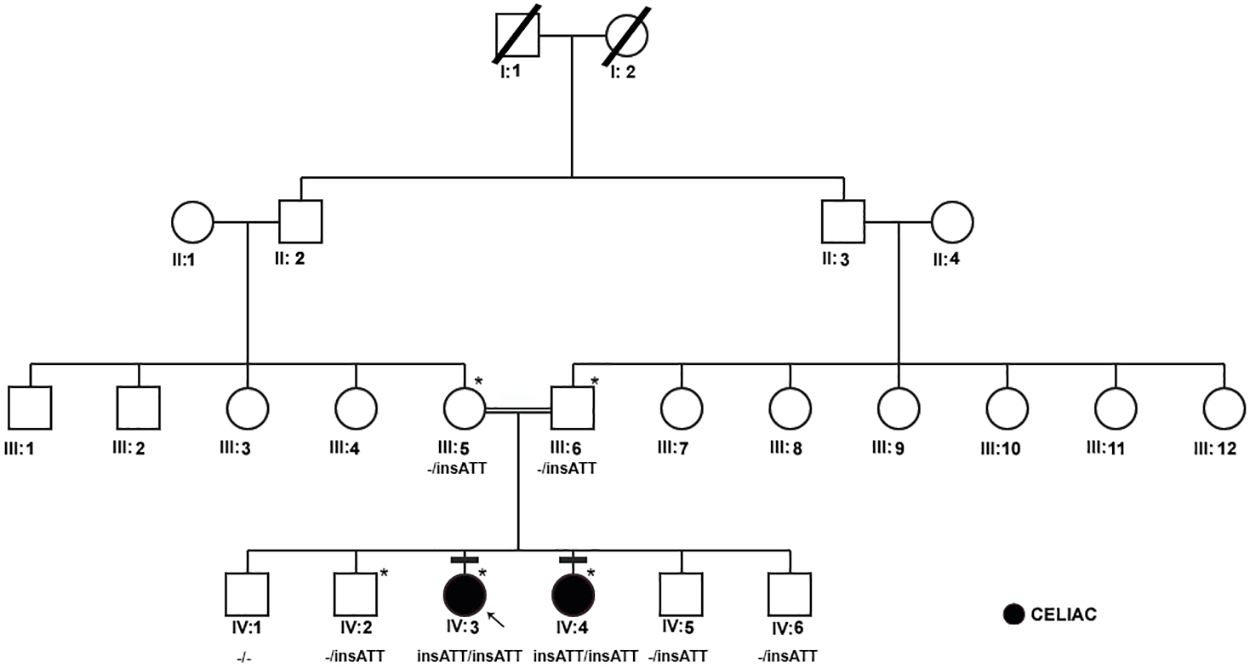
Pedigree analysis of a three-generation consanguineous family presenting with autosomal recessive inheritance of celiac disease. Exome sequenced individuals are indicated with an * mark.

This celiac family [Fig 1] has self-reported that their ancestors (at least 4 generations above) are all of Saudi Arabians by birth and were relatively free from the classic symptoms indicative of celiac disease. In this family, proband IV-3 (21 years old now) was initially diagnosed with type-1 diabetes mellitus (at 9 years of age) when presented with polyuria, polydipsia, and ketoacidosis. Her subsequent screening for endomysial antibodies (at 13 years old) turned to be positive. The duodenal biopsy showed marked infiltration, villous atrophy, and crypt hyperplasia with intraepithelial lymphocytes compatible with the diagnosis of CD (Marsh 3b). She had no gastrointestinal symptoms, and her clinical examination was unremarkable. As part of family screening for CD, serological testing with endomysial antibodies identified that her younger sister i.e. proband IV-4 (at 11 years of age) is also positive. Subsequent small bowel biopsy confirmed the diagnosis of CD with evidence of marked crypt hyperplasia, intraepithelial lymphocytes and villous atrophy (Marsh 3b). She had only mild abdominal pain but no other complaints. Her growth and physical examination were unremarkable. She responded to the gluten free diet and repeated biopsy after 3 years of gluten elimination was normal. The serological screening and clinical studies of unaffected siblings and parents (IV-1, 2, 5 & 6) confirmed that they are free from any kind of autoimmune diseases and did not have any symptoms.

### Genetic analysis

#### Whole exome sequencing: Raw variant data and prioritization

We performed exome capturing and whole exome sequencing of 5 individuals in a CD family, including two CD cases (IV-3 and IV-4), and one of their healthy sibling (IV-1) in addition to both parents (III-1 & III-2). The enrichment of ∼35 Mb of coding sequence has yielded approximately 15 GB of data for each individual. The median exome coverage was about 100 fold with 96% target region. An average of 47,851,116 million reads per exome was identified in the coding regions. At least 70,000 variants per person have met the quality control criteria (minimum Q call was set at 30; minimum depth was set at 30). We found at least 3400 (4.8%) novel variants belonging to missense, nonsense, splice variants and indel categories in each exome (Table 1). From this data set we have selected variants based on their location—exonic, unknown—novel, very rare (minor allele frequency is <0.005%), and by their type—missense and indels. Whereas, non-coding variants (intronic, intergenic), coding region variants, (synonymous or high-frequency alleles with an MAF of >0.005%), regulatory region variants (UTRs and splice sites) were excluded. All mutations which fulfilled the above-mentioned inclusion criteria were filtered based on their functional annotation which reflects the deleterious nature of the variant.

**Table 1:** The genetic variants yield of a celiac disease family.

	Family Member	Total	Homo Alternate	Heterozygote	Novel	Known	Filtered variants
							
**Father**	**III:5**	71734	31727	40007	3516	68218	1146
**Mother**	**III:6**	72756	33498	39258	3697	69059	2533
**Affected 1**	**IV:3**	73882	34767	39115	3847	70035	1189
**Affected 2**	**IV:4**	71569	34591	36978	3598	68027	1120
**Healthy**	**IV:5**	71625	34251	37104	3427	71283	2439

Note: Filtering criteria for variants: exonic, unknown or extremely rare (MAF = <0.005%), type (missense, frame-shifts, indels, UTRs and splice sites).

#### Familial inheritance model and population penetrance

**Fig 2:**
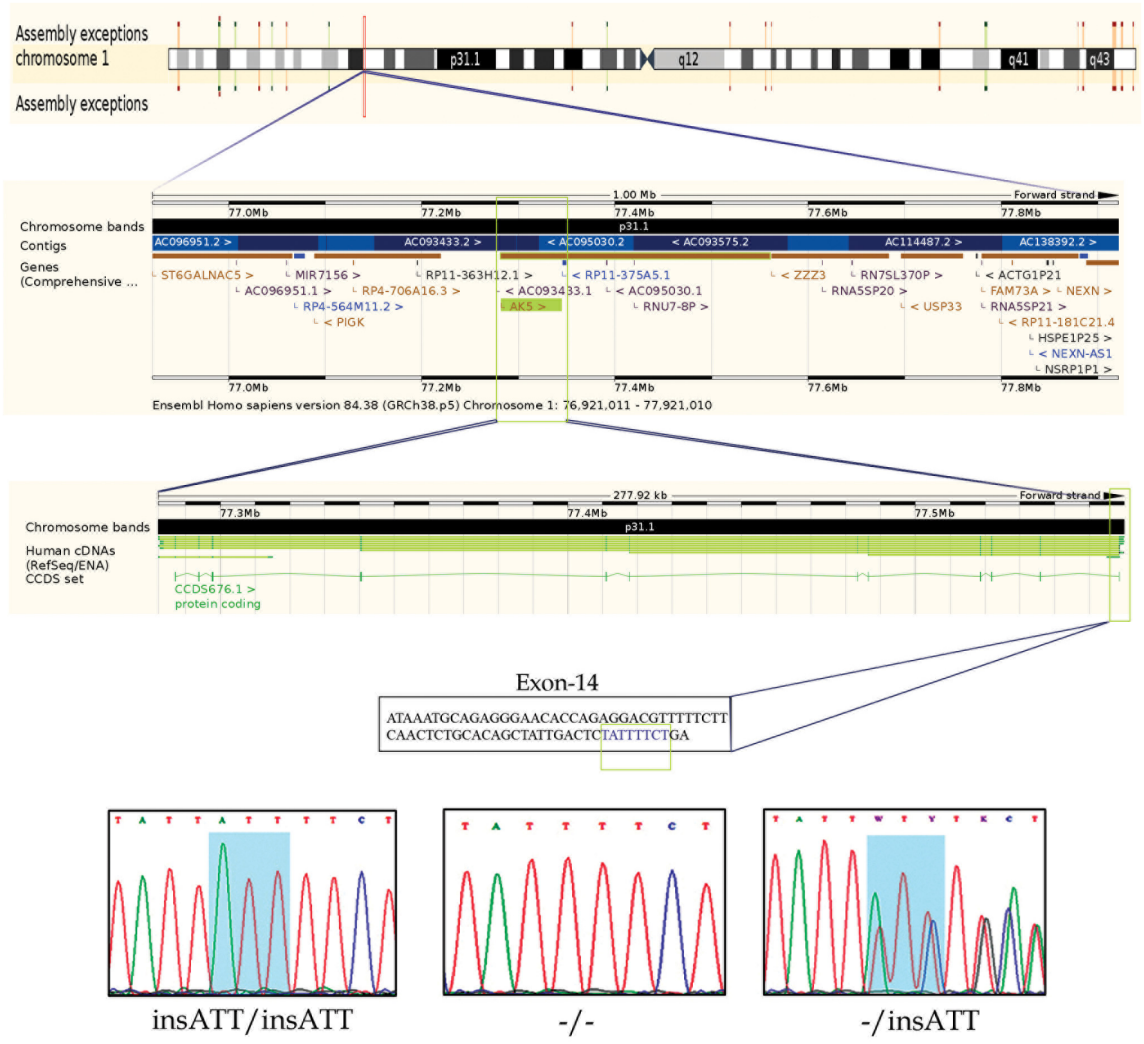
Chromosomal location of human *AK5* gene at 1p31.1 and chromatograms of ATT insertion sequence (wild type, heterozygote, and homozygous mutant genotypes) observed in celiac patients. (Chromosome and gene location figure generated from Ensembl browser).

We analyzed the exome data of this CD family to discover mutations showing either autosomal recessive (AR) or compound heterozygote (CH) inheritance modes. In AR inheritance model, the proband is expected to show the causal allele in homozygote form, given that their parents are heterozygote carriers and siblings could be either homozygous (for the normal allele) or heterozygous. In CH model, two different defective alleles (one from each parent) of the same gene are inherited as compound heterozygotes in the proband. For above-mentioned inheritance patterns, all the variations (exonic, splicing, non-synonymous, frame shift mutations) which had a high population frequency (MAF>0.05) and non-deleterious nature (SIFT score > 0.05 and PolyPhen score < 0.5 predictions) were excluded from further analysis. To our surprise, we could not find any particular gene whose mutation could fulfill the CH inheritance model in this family. But we found 8 genetic mutations (listed in Table 2) which showed classical AR segregation (in exome data only) in this family. However, Sanger sequence validation of all these mutations in the remaining individuals of the family showed that only a 3 bp insertion (ATT) located at nucleotide position 77558664 on chromosome 1 in *AK5* gene has perfectly segregated as per classical AR model (Figs 1 and 2). The underlying reason for genotyping sporadic CD cases and healthy controls is to ascertain if this variant is indeed causal to familial CD (Table 1). However, contrary to our expectation, 45% of the healthy controls and 32% CD cases have the homozygous *AK5* (c. 1683_1684insATT) mutation suggesting its high penetrance in Saudi Population (Table 3). Interestingly, homozygous *AK5* (c.1683_1684insATT) mutation is shown to modify the risk of the healthy Saudi population against CD development (p<0.002). Allelic distribution analysis has also demonstrated the remarkable differences of ATT insertion between our CD cases and healthy controls (p<0.0004).

**Table 2:** List of nucleotide variants from exome data which showed autosomal recessive inheritance model in consanguineous celiac disease family.

S.No	Gene	Chromosome Position	Reference/Alternate Bases	Exonic Region	cDNA Position	Amino Acid Position	dbSNP135_ full	ExAc MAF	GMAF
**1**	VSIG10	12:118506348–53	CTCCTC/-	Exon-8	c.1396_1401del	p.466_467del.	NAv	-	-
**2**	*AK5*	01:78024349–49	-/c.1683_1684insATT	Exon-14	c.1683_1684insATT	p.I561delinsII.	rs76976178	-	-
**3**	WNK2	09:96051100–100	C/T	Exon-19	c.C4064T	p.S1355L,	rs41296061	-	0.0044
**4**	DOK3	05:176930176–178	AGG/-	Exon-5	c.861_863del	p.287_288del.	NAv		
**5**	GNAL	18:11689680–680	-/ TGGCCC	Exon-1	c.118_119insTGGCCC	p.P40delinsLAP.	NAv	-	-
**6**	FNDC1	06:159660804–821	CCCGCCGCACGACCACCA/-	Exon-14	c.4436_4453del	p.1479_1485del	rs3842694	-	-
**7**	LCORL	04:17883693–693	-/AC	UTR-3	UTR3	NA	rs66721989	-	-
**8**	PACRG	06:163149189–189	C/T	UTR-5	UTR5	NA	rs9456807	-	0.0044

Note: NA—Not Applicable; NAv- Not available;

**Table 3:** Genotype and allelic distribution of *AK5*,c.1683_1684insATT variant between celiac sporadic patients and controls, as well as their risk prediction for celiac disease.

Gene	Mutation	Genotype/Allele	Control (%)	Cases (%)	X^2^	p	OR (95% CI)
*AK5*	c.1683_1684insATT	00	20 (19.56)	42 (42.55)	Reference		
		01	35 (35.86)	26 (25.53)	7.85	0.005*	2.82 (1.35–5.89)
		11	45 (44.56)	32 (31.91)	9.458	0.002*	2.95 (1.46–5.94)
		00 vs.01+11	20 vs. 80	42 vs. 58	11.32	0.0007*	2.89 (1.54–5.44)
		01 vs. 00+11	35 vs.65	26 vs. 74	1.91	0.16	0.653 (0.35–1.19)
		11 vs. 00+10	45 vs.55	32 vs. 68	3.569	0.05	0.57(0.32–1.02)
		Allele					
		0	75(37.5)	110 (55)	Reference		
		1	125 (62.5)	90 (45)	12.32	0.0004*	2.03(1.36–3.03)

Wild type Allele = 0; Mutant Allele = 1;

* Indicates a statistical significance

### Computational analysis of AK5 c.1683_1684insATT mutation

**Fig 3:**
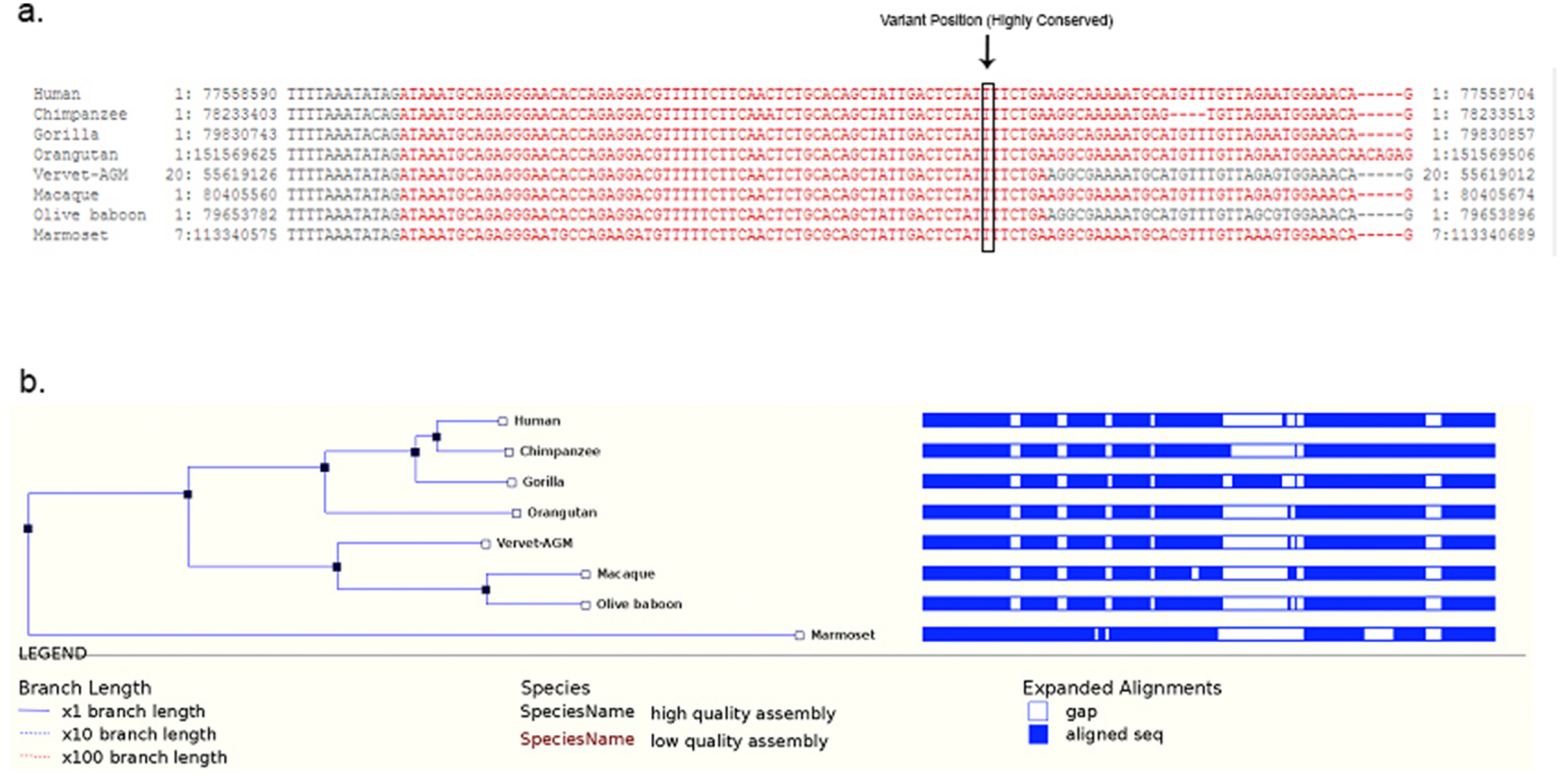
(A) Nucleotide sequence Alignment of human and primate adenylate kinase 5 genes. (B) Phylogenetic tree of the human adenylate kinases.

**Fig 4:**
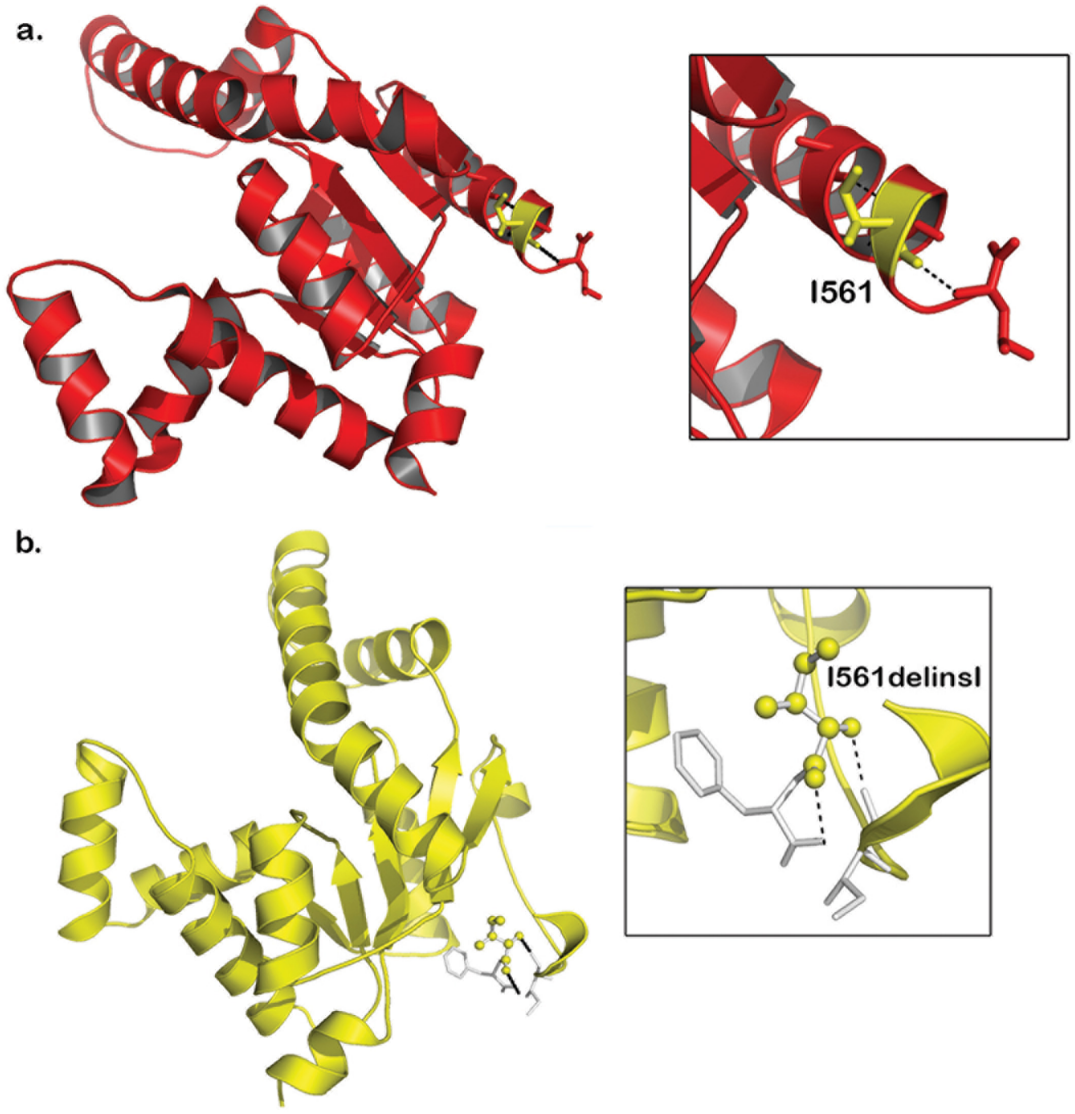
Molecular view of wildtype (Red in color) and mutant (Yellow in color) AK5: a) Hydrophobic bonds arrangement in AK5-wildtype residues Iso561 with Iso556 and Ala557 and b) AK5-mutant residues Iso562 and Phe563.

The nucleotide conservation analysis showed that the c.1683_1684insATT region is highly conserved in humans and also in 7 primates i.e. Chimpanzee, Gorilla, Orangutan, Vervet-AGM, Macaque, Olive Baboon, Marmoset (Fig 3). The Insertion of ATT in the coding region of *AK5* results in the addition of Isoleucine at 561^th^ position of protein. This Isoleucine insertion, in turn, disturbs actual hydrophobic bonds existing in between Isoleucine amino acid (located at 556^th^ position) and Alanine amino acid (located at 557^th^ position) residues lying near the 2^nd^ functional domain of adenylate kinase. However, the structural divergence between native and mutant *AK5* forms is found to be minimal (RMSD value difference is 0.059 Å) (Fig 4).

## Discussion

The advances in sequencing technologies have helped our understanding of auto-immune diseases, specifically by expanding our ability to capture both germ line and *de-novo* mutations [36]. In particular, both candidate gene and GWAS studies have enabled us to understand how genetic variations influence celiac disease risk among sporadic patients [37] [13]. However, these studies have not been able to identify a causative gene so far. In contrast, studying a consanguineous family using the next generation sequencing methods presented us with an opportunity to identify disease segregation in single gene model. So far, two exome studies have been published on celiac disease families. In the first exome study, Szperl AM et al., (2011) [38] have identified 3 linkage regions on 10q23.1-10q23.32, 4q32.3-4q33, and 8q24.13-8q24.21 which showed perfect segregation in a Dutch Caucasian family presenting an autosomal dominant mode of inheritance. The follow-up exome sequencing of 2 affected sibs from this family could not identify any mutant gene from these candidate regions. In a separate investigation, exomes of 75 celiac individuals of European ancestry from 55 families (singletons and multiple) revealed the gene burden of celiac disease amongst closely related subjects [39]. Though they have identified multiple rare coding region variants, none was found to be a causal mutation. Consanguineous celiac families in Saudi Arabia present a potential opportunity to identify rare genetic mutations owing to their large family size and tribal culture [20].

In this report, we presented our experience of working with exome data of a consanguineous family from Saudi Arabia with celiac disease. This study highlights how lack of a population-specific genomic data could easily mislead geneticists from discovering the true positive disease mutations. Our basic intention of the present study was to discover rare and deleterious mutations in this family for celiac disease. The default criteria we set for rare (alternate) alleles, is that they should have a frequency of = <0.005 as per ExAC (Exome Aggregation Consortium) database, which hosts the whole exome data of 60,706 unrelated individuals of various racial groups from different parts of the world [40]. In the context of our study objective, we found insATT in *AK5* gene that is perfectly segregated as an autosomal recessive marker in this consanguineous family with celiac disease.

The *AK5* (adenylate kinase) gene, located at 1p31.1, spanning over 278 kb consists of 14 exons encoding a protein kinase that regulates phosphorylation state of intracellular adenine nucleotide. The 562 aa long *AK5* protein consists of two functional domains, i.e. *AK5*p1 domain (between 137 to 294 aa) and *AK5*p2 domain (between 381 to 537 aa) [41] [42]. Adenylate kinase influences metabolic signals by regulating gene expression levels, activity of cell membrane channels (e.g., ion channels), and also by transferring phosphoryl groups between adenine nucleotides (AMP, ADP, and ATP) [43]. Inherited mutations in *AK5* have been implicated to be the cause of its enzyme deficiency in patients with hemolytic anemia [44]. Involvement of AK5 is also seen in neuronal destruction (through T-cell mediated reactions) among patients of limbic encephalitis, another autoimmune disease [45].

Owing to the known role of *AK5* gene in other T-cell dysfunction related diseases and the fact that CD is a T-cell-mediated autoimmune disorder, we initially believed its causal role in CD development in the consanguineous family we investigated. However, the surprise finding of increased prevalence of a globally extremely rare (MAF is 0.000008) c.1683_1684insATT insertion in *AK5* in healthy Saudi population (MAF is 0.63) rules out it as a possible genetic cause of familial CD. Although rare recessive mutations are expected to be present in every ethnic population, they rarely achieve homozygosity in mixed populations, as they do in consanguineous populations [46]. Given the high consanguineous background rate in Saudi Arabia, we assume that the homozygous form of *AK5* c.1683_1684insATT variant is positively enriched in the healthy population due to differential selection pressures. The positive selection increases the prevalence of adaptive traits by increasing the frequency of possibly favorable alleles like AK5 c.1683_1684insATT. Owing to the importance of multiple genetic and environmental factors for the development of CD, it is speculated that the individuals with rare AK5 allelic variant resist the disease development through unknown complex gene-environment interactions. In this context, the significant difference in insATT allelic frequency distribution among healthy controls and sporadic CD cases has led us in proposing this variant as a possible risk modifier against the development of CD.

Our hypothesis gains additional support from computational structure studies, which predicts that the insertion of Isoleucine at 561^st^ position of *AK5*, shifts the actual hydrogen bond existing between tyrosine amino acid (located at 400^th^ position) and aspartic amino acid (located at 556^th^ position) residues lying in the 2^nd^ functional domain of adenylate kinase. This shuffling of amino acids and their interactions is likely to impact the secondary structural orientation of the polypeptide, eventually inducing the gain-of-function in the nucleoside phosphate kinase activity of *AK5* protein, which would then modulates ADP/ATP ratio in intestinal cells. The ATT insertion mutation of *AK5* may also disturb the homeostasis of ATP/GTP and lowers tissue transglutaminase levels (regulated by cytosolic guanine–adenine nucleoside concentrations). The lower tissue transglutaminase levels simultaneously lead to the reduced gluten peptide deamidation and may compromise its presentation by dendritic cells to mucosal CD4+ T-cells in the context of their positivity to HLA-DQ2 and HLA-DQ8 status. Therefore, it is reasonable to assume that *AK5* c.1683_1684insATT insertion indirectly down-regulates the gluten reactivity of CD4+ T-cells and negatively affects the pro-inflammatory cytokine levels, thus Th1 response, eventually modifying the risk of individuals against developing typical crypt hyperplasia, intraepithelial, celiac lesion-villous atrophy and lamina propria infiltration of inflammatory cells. Although genetically susceptible background is essential, CD occurrence greatly depends on the interaction of genetic and environmental factors including gut microbiota in individuals [47].

We acknowledge some limitations in the present study. Our exome data filtration was done using the genomic data of Caucasian, Asian and African populations. Lack of Arab population-specific genomic data misled us in selecting a locally common variant (but globally rare) of Arab population as a potential causal candidate for the CD in the consanguineous family, we studied. By combining genome-wide homozygosity mapping procedures and whole-exome sequencing, our colleagues were able to identify a recurrent homozygous missense mutation (c.899G>A; p.Gly300Asp) in exon 7 of cathepsin C gene in a consanguineous family with Papillon–Lefevre syndrome [48]. But our strategy did not include the homozygosity mapping technique, which could have potentially helped us to focus on some loss of heterozygosity (LOH) regions due to the consanguineous marriage of the parents.

In conclusion, through exome sequencing of a Saudi celiac family, we have found a rare *AK5* +ATT insertion mutation showing the autosomal recessive mode of inheritance. However, the genetic etiology of CD in this family is more complicated than the classic autosomal recessive inheritance pattern we have initially led to believe and expect. The experience obtained from this study suggests that WES alone may not be able to identify disease causal genes of celiac disease showing familial presentation. To zoom-in on the CD-causal genes, the WES results need to be carefully interpreted in combination with the data sets generated from homozygosity mapping, gene expression, epigenetics and gut microbiome experiments. Our study underlines the need to have population-specific databases of variants, generated through large scale next generation sequencing projects across the Middle East to address the disease marker identification. Thus to derive successful outcomes from exome sequencing of rare complex disease families in Saudi population, we recommend using many consanguineous families, Arab validation cohorts (sporadic cases and controls) and the development of unique Arab reference database.

## Supporting information

S1 Table Primer sequences of prioritized gene list from exome celiac study. (PDF)
