# Supplementary material for: Whole exome sequencing of a consanguineous family identifies the possible modifying effect of a globally rare AK5 allelic variant in celiac disease development among Saudi patients
Source: PLoS One. 2017 May 15;12(5):e0176664. doi: 10.1371/journal.pone.0176664 (PMC5432167; doi:10.1371/journal.pone.0176664)
Supplement: S1 Table — (PDF) [file pone.0176664.s001.pdf]

S1 Table: Primer sequences of prioritized gene list from exome celiac study

S.No	Gene	Chromosome Position	Ref/Alternate Bases	Primer (Forward(F): Reverse (R))	
**1.**	AK5	01:78024349-49	-/ ATT	GCAGAGCAAATTATG AGC (F)	TAGCTGGGAAGCAAA CAGT(R)
**2.**	VSIG10	12:118506348-53	CTCCTC/-	TCAGTGAGGGCTGAA GTTGG(F)	AAGGCGGTCACTCTG TGAAT(R)
**3.**	WNK2	09:96051100-100	C/T	GTCGGGAAACAGAGA AGACCA(F)	GAGCTGCTGACTGGC TAGAAA(R)
**4.**	DOK3	05:176930176-178	AGG/-	AACCGATGACGTATC AGGCA(F)	AGGTTCAGGATGTGC CAAGG(R)
**5.**	GNAL	18:11689680-680	-/ TGGCCC	GGGTCTGTGCTACAG TCTGC(F)	GCTTCTCCTTCGGCTT GTCT(R)
**6.**	FNDC1	06:159660804-821	CCCGCCGCA CGACCACCA/-	TGAGTCTTGGAGGAA AGC(F)	TCAGGTTGCCATCATC GT(R)
**7.**	LCORL	04:17883693-693	-/AC	GAACACTGAAGACTC CTCCGA(F)	CACACAAATACACAC CTG(R)
**8.**	PACRG	06:163149189-189	C/T	TGGAAGCTTGTTGCA GCTCT(F)	TTCAGGAAGATCACT TACGA(R)
